# BRAF V600E mutation in early-stage multiple myeloma: good response to broad acting drugs and no relation to prognosis

**DOI:** 10.1038/bcj.2015.24

**Published:** 2015-03-20

**Authors:** E H Rustad, H Y Dai, H Hov, E Coward, V Beisvag, O Myklebost, E Hovig, S Nakken, D Vodák, L A Meza-Zepeda, A K Sandvik, K F Wader, K Misund, A Sundan, H Aarset, A Waage

**Affiliations:** 1 KG Jebsen Center for Myeloma Research, Norwegian University of Science and Technology, Trondheim, Norway; 2 Department of Cancer Research and Molecular Medicine, Norwegian University of Science and Technology, Trondheim, Norway; 3 Department of Pathology and Medical Genetics, St Olavs Hospital, Trondheim, Norway; 4 Department of Laboratory Medicine, Children's and Women's Health, Norwegian University of Science and Technology, Trondheim, Norway; 5 Bioinformatics Core Facility, Department of Cancer Research and Molecular Medicine, Norwegian University of Science and Technology, Trondheim, Norway; 6 Norwegian Cancer Genomics Consortium, Norway; 7 Department of Tumor Biology, Institute for Cancer Research, Radium Hospital, Oslo University Hospital, Oslo, Norway; 8 Institute for Cancer Genetics and Informatics, Radium Hospital, Oslo University Hospital, Oslo, Norway; 9 Biomedical Informatics group, Department of Informatics, University of Oslo, Oslo, Norway; 10 Bioinformatics Core facility, Institute for Cancer Research, Radium Hospital, Oslo University Hospital, Oslo, Norway; 11 Genomics Core facility, Institute for Cancer Research, Radium Hospital, Oslo University Hospital, Oslo, Norway; 12 Department of Oncology, St Olavs Hospital, Trondheim, Norway; 13 Department of Hematology, St Olavs Hospital, Trondheim, Norway

## Abstract

In this study, we analyzed the prevalence and clone size of BRAF V600E mutation in 209 patients with multiple myeloma and related the results to clinical phenotype, response and survival. Biopsies were screened for BRAF V600E by allele-specific real-time PCR (AS-PCR). Positive results were confirmed by immunohistochemistry, Sanger sequencing and, in three patients from whom we had stored purified myeloma cells, whole-exome sequencing. Eleven patients (5.3%) were BRAF V600E mutation positive by AS-PCR and at least one other method. The fraction of mutated cells varied from 4 to 100%. BRAF V600E-positive patients had no characteristic clinical phenotype except for significantly higher levels of serum creatinine (125 versus 86 μmol/l) Seven of eleven patients responded with at least very good partial response to alkylators, immunomodulatory agents or proteasome inhibitors. Progression-free and overall survival were similar in patients with and without the mutation. By this integrated approach, we found that patients with BRAF V600E mutation responded very well to broad acting drugs and there was no relation to prognosis in early-stage myeloma. In particular, a large mutated cell fraction did not correlate with aggressive disease.

## Introduction

The oncogenic BRAF V600E mutation causes constitutive activation of the Ras-Raf-MEK-ERK (RAS) signaling pathway, stimulating cellular growth, differentiation and survival.^1^ Although rare in multiple myeloma, this mutation has attracted attention because of its proven potential for targeted inhibition. In metastatic malignant melanoma, which harbors BRAF V600E in 35–41% of cases,^2, 3^ treatment with the small-molecular BRAF V600E inhibitor vemurafenib has resulted in improved overall survival (OS) in a phase III clinical trial,^4^ although the long-term benefit was limited by the rapid acquisition of resistance. Promising results from BRAF V600E inhibition have also been seen in patients with various other cancers harboring BRAF V600E. These include anaplastic thyroid carcinoma,^5^ pulmonary adenocarcinoma^6^ and hairy cell leukemia.^7^

The clinical significance of BRAF V600E in multiple myeloma has been characterized in one study.^8^ Seven myeloma patients with BRAF V600E had significantly shorter OS (45 versus 105 months) and increased incidence of extra medullary disease (EMD; 57% versus 17%) compared with 251 patients with wild-type (wt) BRAF. They also reported targeting BRAF V600E in one patient who achieved a durable remission by vemurafenib. Since then, three additional cases of refractory myeloma with BRAF V600E mutation being treated with vemurafenib have been reported.^9, 10^ Two patients had short durations of response, whereas the third still had ongoing response 4 months after initiation of therapy.

Lohr *et al.* recently published a genome sequencing study of 203 multiple myeloma patients, highlighting the vast genetic heterogeneity of this disease.^11^ BRAF V600E appeared in both major and minor clones, but rarely in the entire tumor cell population.^11, 12^ When a myeloma patient is exposed to various treatment regimens, a changing and unpredictable pattern of clonal resistance and dominance may occur.^13, 14^ It has been suggested that myeloma subclones harboring BRAF V600E might have a survival advantage, and that once the BRAF V600E clone becomes dominant, the disease becomes more aggressive.^8, 9^

The genetic heterogeneity and changing clonal dominance of multiple myeloma poses a challenge in defining the conditions for application of targeted therapy. Although exposing BRAF-mutated myeloma cell lines to BRAF inhibition *in vitro* causes a reduction in RAS-pathway activity, the opposite effect is seen in BRAF wt cells, especially when a RAS mutation is also present.^11^ This paradoxical effect indicates that BRAF inhibitor treatment may be harmful in patients with small BRAF V600E-mutated subclones, underlining the need of accurate characterization of candidates for BRAF inhibitor treatment. Furthermore, it is also indicated that mutated BRAF should not be targeted in patients with cells harboring mutated RAS.^15^

Only 10 myeloma patients with BRAF V600E have been described so far: 7 in a retrospective study and 3 case reports. The biological and clinical significance of this mutation is by no means clarified. In this retrospective study, we have analyzed biopsies from 209 patients with myeloma, 11 of whom harbor the BRAF V600E mutation. In particular, we wanted to examine patients carrying the BRAF V600E mutation and their relation to clinical phenotype, treatment response and survival.

## Materials and methods

### Patient selection and data collection

The database at the Department of Pathology and Medical Genetics was searched for biopsies classified as multiple myeloma or plasmacytoma between 1996 and 2012, identifying biopsies from 209 patients with multiple myeloma (*n*=196) or smoldering myeloma (*n*=10) as defined by the International Myeloma Working Group (IMWG) criteria.^16^ In three cases, it was not clear whether the patient had symptomatic disease. One hundred and seventy-three patients (88.3%) were diagnosed with symptomatic myeloma after 2000 when treatment with new drugs (thalidomide) was introduced. A total of 188 patients had only bone marrow biopsies, 14 only had a biopsy from extramedullary lesions, while 7 patients had both. Biopsies from 185 patients were obtained before a relapse had occurred. From 22 patients, the biopsy was obtained later in the course of disease. Time of biopsy was missing for two patients.

Patients who were alive at the time of inclusion were informed about the study by a letter and given the option to withdraw. The Regional Ethics Committee approved the study (REK 2165-2012).

OS was calculated from start of treatment until death. Progression-free survival (PFS) was calculated from start of treatment until death or progressive disease, whatever came first. Presence of EMD was evaluated based on patient records, imaging and biopsy descriptions. EMD was classified in two groups: those extending from the bone marrow and those growing independent of the bone marrow.^17^ Treatment response was evaluated according to IMWG criteria.^18^ If serum M-protein was not reduced by at least 50% following treatment (partial response, PR), the patient was considered resistant to the drugs in question. Acquired resistance developing after initial sensitivity for a specific drug was not included in the evaluation.

### BRAF mutation analysis

All biopsies were screened for BRAF V600E and BRAF K601N by PCR. Biopsies with positive screening were examined by Sanger sequencing and immunohistochemistry (IHC). Whole exome sequencing (WES) was conducted in three patients from whom we had previously isolated and stored CD138^+^ bone marrow cells in our myeloma biobank. Patients were classified as BRAF V600E positive when the mutation was detected by allele-specific real-time PCR (AS-PCR) and confirmed by at least one other detection method.

### Isolation of genomic DNA and mutation analysis of BRAF

Genomic DNA from sections of bone marrow biopsies of myeloma patients was isolated using QIAamp DNA FFPE Tissue Kit (Qiagen, Venlo, Netherlands) according to the manufacturer's instruction. DNA concentration was assessed by NanoDrop spectrophotometer (Thermo Fisher Scientific, Waltham, MA, USA). AS-PCR for BRAF V600E and BRAF V600 wt was performed using a Bio-Rad DNA Engine Opticon 2 Real-Time Cycler (Bio-Rad Laboratories, Hercules, CA, USA). The reaction mix consisted of 1XGeneAmp PCR Gold buffer (Applied Biosystems, Thermo Fisher Scientific), MgCl_2_ (1.5 mM), dNTP (0.4 mM), 6 μM of each primer, Taqman probe (0.2 μM) and 1 U AmpliTaq Gold DNA polymerase, in a total volume of 25 μl. The amount of 20 ng genomic DNA was applied for each PCR reaction with the following program: denaturation at 94 °C for 10 min followed by 40 cycles of incubation at 94 °C for 30 s and then at 60 °C for 30 s. AS-PCR was performed for BRAF V600E mutation detection with following sense primer for BRAF V600: 5′-TAGGTGATTTTGGTCTAGCTACTGT-3′ BRAF V600E: 5′-TAGGTGATTTTGGTCTAGCTACTGA-3′ and antisense primer: 5′-CCACAAAATGGATCCAGACA-3′ and Taqman probe: 5′-TCGATGGAGTGGGTCCCATCA-3′. AS-PCR was performed for BRAF K601N mutation detection with sense primer: 5′-AAACTCTTCATAATGCTTGCTCTG-3′ and antisense primer: 5′-GGACCCACTCCATCGACAT-3′ (K601) and 5′-GGACCCACTCCATCGACAA-3′ (K601N).

The sensitivity of BRAF K601N detection was at least 1% and was tested with BRAFK601N-positive cell line U266 in the background of BRAF V600 wt bone marrow biopsy. All BRAF V600E-positive samples shown by AS-PCR were retested and reproduced. Sanger sequencing of BRAF exon 15 was used to verify BRAF V600E detected by AS-PCR. The sequence was analyzed with a 3130 ABI capillary sequencer (Applied Biosystems).

### Immunohistochemistry

IHC was carried out on formalin-fixed paraffin-embedded samples, using the mouse monoclonal anti-BRAFV600E antibody (clone VE1, Springer Bioscience, Pleasanton, CA, USA). Slides were stained in a Dako Autostainer Link 48 (Dako, Glostrup, Denmark) in a standard procedure using the EnVisionTM FLEX+, Mouse, High pH (Link) kit from Dako. Briefly, freshly cut 4-μm sections of formalin-fixed paraffin-embedded tissue blocks were pretreated with Target Retrieval Solution High (pH 9) for 20 min at 85 °C and incubated for 40 min with a 1:100 dilution of the primary antibody. The procedure included a secondary reagent and a HRP-labeled polymer. 3,3′-Diaminobenzidine was used for chromogenic detection. Slides were subsequently washed in water, incubated with 0.5 % (w/v) copper sulphate for 3 min and counterstained with hematoxylin for 15 s. The antibody was tested out on formalin-fixed paraffin-embedded samples from AS-PCR BRAF V600E-positive and -negative malignant melanoma, adenocarcinoma of the colon, and hairy cell leukemia. Sections from the myeloma patients with positive AS-PCR were evaluated independently by two hematopathologists. As the work progressed, we realized that the percentage of BRAFV600E-positive plasma cells varied substantially between patients. We therefore estimated the proportion of positive plasma cells in quartiles compared with slides stained with antibodies against CD138, or kappa or lambda light chains. Sections from 20 myeloma patients with BRAF V600E-negative AS-PCR were investigated for BRAF V600E by IHC. They were all found negative.

### Patient myeloma cells and mononuclear cells

CD138-positive myeloma cells were isolated from bone marrow samples obtained from the Norwegian Myeloma Biobank, using a RoboSep automated cell separator and a Human CD138 Positive Selection Kit (StemCell Technologies, Grenoble, France).^19^ The purity of plasma cells after isolation was above 90%. Mononuclear cells were isolated from whole blood using a Vacutainer cell preparation tube with sodium citrate (BD, Franklin Lakes, NJ, USA) according to the manufacturer's instructions.

### Whole-exome sequencing

DNA was extracted from mononuclear blood cells and purified CD138 cells from bone marrow using QIAamp DNA Mini Kit (Qiagen, Germantown, MD, USA) according to the manufacturer's instructions. Exome sequencing libraries were prepared from 1 μg genomic DNA using the SureSelectXT target enrichment system for Illumina paired-end sequencing libraries (Agilent Technologies, Santa Clara, CA, USA), according to the manufacturer's instructions. Briefly, the DNA was fragmented using the Covaris M220 system (Covaris, Woburn, MA, USA). The DNA fragments (183±3 bp) were end-repaired using T4 DNA polymerase, Klenow DNA polymerase and T4 polynucleotide kinase (PNK), followed by purification using AMPure XP beads (Beckman Coulter, Brea, CA, USA). Fragments were A-tailed using Klenow DNA polymerase and dATP and purified using AMPure XP beads. Indexing adapters for sequencing were ligated to the DNA fragments, followed by purification using AMPure XP beads. The adapter-ligated libraries were amplified for six PCR cycles, followed by a second purification using AMPure XP beads. The quality of the enriched libraries was evaluated using the 2100 Bioanalyzer and a DNA 1000 kit (Agilent Technologies, Santa Clara, CA, USA) and quantitative PCR. Exon capture was performed from 1 μg of each sequencing library using the SureSelectXT Human All Exon V5 target 50 Mb kit (Agilent Technologies). Briefly, the fragments in the library were hybridized to capture probes (20 h at 65 °C), unhybridized material was washed away and the captured fragments were amplified for 10 PCR cycles, followed by purification using AMPure XP beads. The quality of the enriched libraries was evaluated using the 2100 Bioanalyzer and a High-Sensitivity DNA-kit (Agilent Technologies). The adapter-ligated fragments were quantified by quantitative PCR using the KAPA SYBR FAST library quantification kit for Illumina Genome Analyzer (KAPA Biosystems, Woburn, MA, USA). A 20 pM solution of the sequencing libraries was subjected to cluster generation on the cBot instrument (Illumina, Inc., San Diego, CA, USA). Paired-end sequencing was performed for 2 × 100 cycles on a HiSeq2500 instrument (Illumina, Inc.), according to the manufacturer's instructions.

Base calling was done on the HiSeq instrument by RTA 1.17.21.3. Fastq sequence files were generated using CASAVA 1.8.2 (Illumina, Inc.).

### Sequence analysis and somatic variant calling

Paired-end sequencing reads of length 100 bp were aligned to the human reference genome (hg19, without haplotype chromosomes and unplaced contigs) using the Novoalign tool (version 2.08.03, http://www.novocraft.com). Novoalign was run with custom options set for expected pair-end insert size correction (‘-i PE 220,60') and multiple mapper reporting (‘-r All 10'). Sequencing reads that did not map properly to the genome (that is, no match, multiple matches, paired reads matching different chromosomes, or paired reads matching the same orientation) were excluded from the analysis. PCR-derived duplicate reads (judged by mapping location and nucleotide sequence) with lowest sum of base qualities were also discarded. Re-alignment was performed around indels with GATK RealignerTargetCreator and IndelRealigner tools (version 2.3.9-ge5ebf34), followed by an application of Picard FixMateInformation (http://picard.sourceforge.net, version 1.84).^20^ Base quality recalibration was done with GATK BaseRecalibrator. Among the tumor samples, we obtained a minimum sequencing coverage of 100 × for 85.7% of the exonic-targeted regions. Similarly, for the matching control samples, a minimum sequencing coverage of 50 × was obtained for 87% of the exonic-targeted regions.

MuTect (version 1.1.4) was applied on the matched tumor-control alignments to identify somatically acquired point mutations.^21^ MuTect employs a Bayesian classifier to detect somatic mutations with very low allele fractions, requiring only a few supporting reads, followed by carefully tuned filters that ensure high specificity. To minimize the number of potentially false positive variants, all variants that passed MuTect filters were in addition subject to the following hard filters: a minimum sequencing coverage of 14 × at the variant site in the tumor and a minimum of 5 positive quality reads supporting the alternative allele, a minimum sequencing coverage of 8 × in the control and less than 5% of the reads supporting the alternative allele. An exception from this was made for the BRAF V600E mutation, which was already detected by AS-PCR in all three patients. In this context, the mutation can be considered present with only 2–3 supporting reads in the tumor sample.

To understand the functional role of identified variants, all variants were subject to a computational annotation workflow that included ANNOVAR (released on 23 August 2013, using RefSeq as the gene model), COSMIC (known somatic cancer variants and Cancer Gene Census, v68), PFAM (protein domain information, v27.0), UniProt (functional protein properties, release 2014_05), PROSITE (predicted functional protein sites, Release 20.100 of 19Feburary 2014), the Drug Gene Interaction Database (druggable targets, version 1.63) and dbNSFP (computational predictions of effect of coding variants, v2.4).^22, 23, 24, 25, 26, 27, 28^

### Image processing

Histology images were captured at room temperature with a Nikon eclipse Ci microscope (Nikon Gmbh, Düsseldorf, Germany) with a Lumenera Infinity 2 camera and Infinity analyze software, release 6.2 (Lumenera Corporation, Ottawa, Ontario, Canada).

Images and graphic materials were processed in Adobe Illustrator v 6 and Adobe Photoshop CS6.

### Statistical analysis

OS and PFS in BRAF V600E and BRAF wt groups were compared by Kaplan–Meier estimates and Log Rank test. Differences in clinical parameters were evaluated by *T*-test, Mann–Whitney *U*-test or Fisher's exact test. Level of statistical significance was set at *P*<0.05, and *P*-values were two-tailed. Statistical analysis was carried out by SPSS v. 21 (IBM Corporation, Armonk, NY, USA).

## Results

### Prevalence of BRAF V600E mutation

BRAF V600E was detected by AS-PCR in bone marrow or EMD from 11 out of 209 patients (5.3%) with multiple myeloma (Table 1). Two additional patients with positive BRAF V600E AS-PCR were classified as negative because the mutation was not confirmed by another method. The prevalence of BRAF V600E mutation in EMD was 4.8% (1/21). No BRAF K601N mutations were detected by AS-PCR screening of 190 bone marrow biopsies.

### Evaluation of BRAF V600E-positive clones

**Figure 1:**
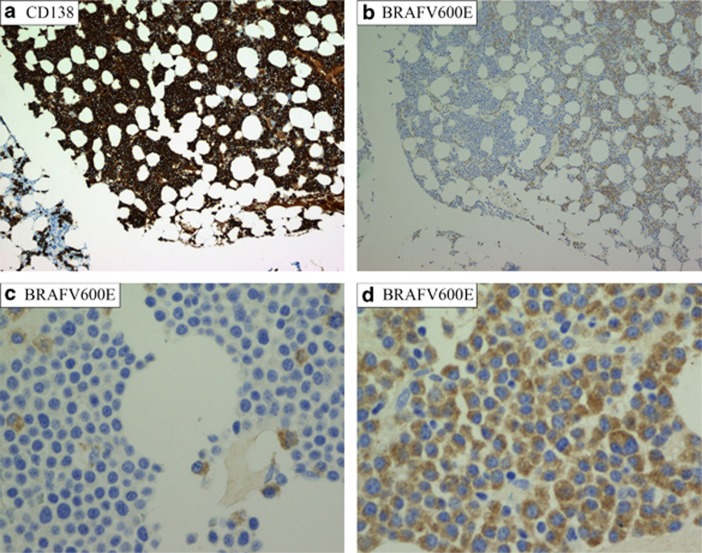
IHC showing fraction and growth pattern of BRAF V600E mutated plasma cells. Microscopic images of bone marrow biopsy from patient 4 (Table 1). (**a**) The bone marrow was in large areas packed with CD138-positive myeloma cells. × 100 magnification. (**b**) The same area as in **a** with the BRAF V600E antibody. On the left side, almost none of the myeloma cells were positive, whereas the mutant clone predominated on the right side. × 100 magnification. (**c** and **d**) High-power field ( × 400) images from the area to the left (**c)** and right (**d**) side in image **a** and **b**. (**c**) Single scattered BRAF V600E-positive myeloma cells among predominating negative myeloma cells. In **d**, almost all myeloma cells were BRAF V600E positive.

The fraction of BRAF V600E-positive cells as estimated by IHC is shown in Table 1. The growth patterns of BRAF V600E-positive cells were different in different areas of the biopsies and differed between patients. The distribution of positive cells was either in confluent sheets or as disseminated cells. In areas where the bone marrow was packed with CD138-positive myeloma cells (Figure 1a), the proportion of BRAF V600E-positive cells varied substantially (Figure 1b) indicating different myeloma subclones. The presence of single scattered positive cells among a predominating negative myeloma clone rules out the possibility of false negative areas because of technical error, fixation differences and so on. It also indicates that these BRAF V600E-positive cells do not possess a growth advantage over the surrounding negative cells in these areas.

Clone size was also evaluated by WES in patients 1, 8 and 11, from whom we had available material (Table 2). Exomes from the myeloma cells and blood mononuclear cells were sequenced at a depth of 100-fold and 50-fold, respectively (for at least 80 % of exons). Clone fraction of BRAF V600E was comparable with that found by IHC in patient 1 who had a dominating clone, whereas the correspondence was not perfect for small clones in patients 8 and 11.

### Additional information from WES

Among the top 11 recurrently mutated genes listed in Lorh *et al.*,^11^ we found mutations only in BRAF, NRAS and KRAS. Combined mutations in BRAF/NRAS or BRAF/KRAS were found in two of three investigated patients. Both had small fractions of BRAF mutated cells (Tables 1 and 2).

No genes with coding mutations after filtering were shared between all three patients. Patients 1 and 11 shared one gene (ATXN1), patients 8 and 11 shared one gene (HCFC2) and patients 1 and 8 shared three genes (IQSEQ3, PCMTD1, PNRC2). For complete list of mutations, see the online Supplementary Information (Supplementary Table 1).

### Clinical characteristics, drug sensitivity and survival of patients with BRAF V600E mutation

Creatinine was significantly higher in the BRAF V600E mutated group (Table 3). Other characteristics were distributed equally or non-significantly different between the two groups. This was also the case for characteristics that might influence outcome, like age, ISS (International Staging System) stage and exposition to newer drugs or high-dose therapy and autologous stem cell transplant (Table 3).

**Figure 2:**
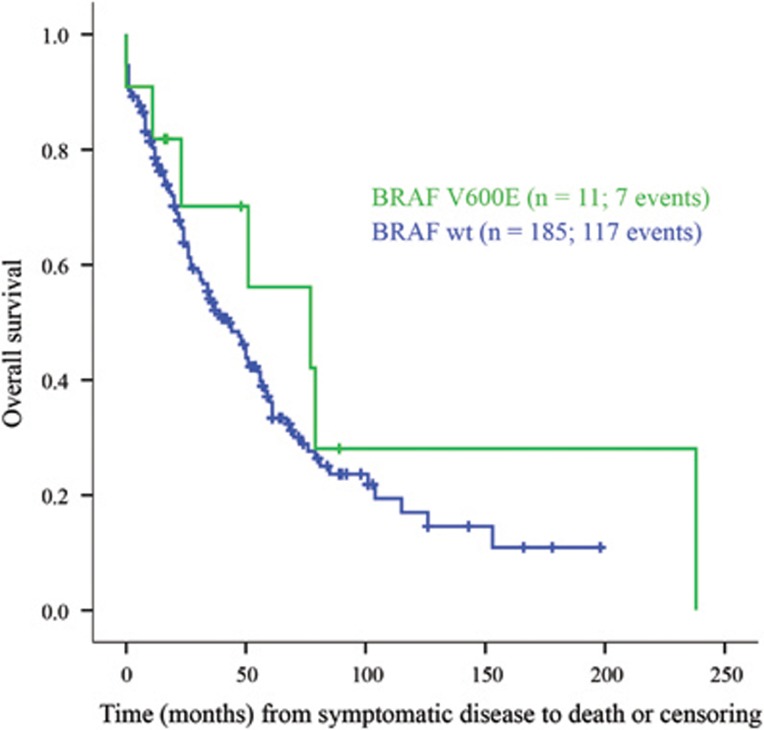
Overall survival in patients with and without BRAF V600E mutation shown by Kaplan–Meier plot.

There was no significant difference in PFS (data not shown) or OS (Figure 2) between the BRAF V600E-positive and -negative groups. The result was the same also for five patients harboring BRAF V600E in more than 50% of the plasma cells. There was no difference in the prevalence of EMD (Table 3).

BRAF V600E-positive patients were sensitive to a variety of treatment regimens (Table 1). Excluding one patient with missing response data, 7 of 10 patients responded with very good partial response (VGPR) or better. All three patients receiving high-dose therapy and autologous stem cell transplant responded. There were sensitivity and resistance to alkylators, immunomodulatory agents and proteasome inhibitors without any particular pattern. Two patients with NRAS or KRAS mutation combined with a small BRAF V600E clone responded with VGPR.

## Discussion

In this study, we have analyzed clone size and growth pattern of BRAF V600E-positive cells related to clinical information, treatment response and survival. Patients harboring BRAF V600E clones of any size responded very well to treatment as 7 of 11 patients achieved VGPR or better to at least one treatment regimen. We conclude that these patients can be highly sensitive to broad acting drugs like alkylators, immunomodulatory agents and proteasome inhibitors.

When there is a >90% reduction of the total clonal cell mass, we can infer that also the V600E-positive cells are sensitive to broad acting drugs in most cases.

We detected the BRAF V600E mutation in 5.3% of patients with multiple myeloma. In 1.4% (3 patients), there was a dominating clone with more than 75% BRAF V600E mutated cells, which can be regarded as a valid rationale for BRAF inhibitor treatment. Translation of the mutated gene to protein, which is another prerequisite for inhibitor treatment, was demonstrated by IHC in 10 of 11 patients. Examples of excellent effect of specific BRAF V600E inhibition have been published.^9, 10^ However, these patients have been in a late stage with acquired resistance to several drugs. Our analysis is based on early-stage disease, which may be very different. Taken together, the clonal heterogeneity that is frequently present and the broad sensitivity of BRAF V600E-positive clones indicate that broad acting drugs still should be a backbone of the treatment regime. If specific BRAF inhibition is applied, there is a rationale to give it in addition to broad acting drugs. The benefit of specific BRAF inhibition in this situation of course needs to be tested separately in a prospective clinical trial.

PFS and OS were equal in patients with and without BRAF mutation V600E. Creatinine was the only factor that was significantly different. There were smaller non-significant differences in ISS stage III and exposition to thalidomide, bortezomib, lenalidomide and carfilzomib, which were more frequent in the V600E group, and for ISS stage II and high-dose therapy and autologous stem cell transplant, which was more frequent in the non-V600E group. The three patients with >75% BRAF V600E mutated clone all had relatively indolent disease courses with OS of 48, 77 and 89 months. We conclude that the presence of BRAF V600E at diagnosis or early in the disease, regardless of clone size, is not correlated with particularly aggressive disease in our cohort of patients. Renal damage was associated with BRAF V600E mutation, but this needs to be confirmed by other studies to establish a true relation. Except for this, we found no characteristic clinical phenotype related to the BRAF V600E mutation. We found no BRAF K601N mutations in the 190 patients screened, which is in line with the previously reported prevalence of 0 and 1.9%.^11, 29^

WES confirmed the presence of the actual BRAF V600E mutation in two of three patients that were analyzed. We should keep in mind that the sequencing was adapted to whole-exome scanning (depth of coverage of approximately 100 × ) and this resulted in a sensitivity inferior to that obtained by AS-PCR and IHC. Lohr *et al.* calculated that they could detect clone sizes down to 10% with a mean coverage of 89 × .^11^ In our study, the three patients were all found to be positive for the BRAF V600E mutation by PCR, and two of them additionally by IHC. This allows for a higher significance level for a statistical evaluation of positive result by sequencing, that is, we are able to detect smaller clones. The lower sensitivity of WES can easily be compensated at a low cost if targeted sequencing of a limited number of genes is carried out.

The clone size as estimated by IHC and WES corresponded well in the patient with a dominating BRAF V600E mutated clone, but less so in patients with a smaller percentage of cells carrying the mutation. Random differences in the sampling and bone marrow composition may explain this difference. Both methods add unique information. IHC visualized differences in the growth pattern of BRAF V600E mutated cells, which appeared in a tumor-like manner or as disseminated cells. Whether this has an association to signaling activity or tumor growth remains to be seen.

Of the top 11 list of recurrent mutations published by Lohr *et al.,*^11^ we only detected mutations in BRAF, KRAS and NRAS. RAS mutations are of particular interest because *in vitro* studies indicate that their presence may imply a paradoxical effect of BRAF inhibitors. This effect is not yet demonstrated in patients, but should be taken into consideration if BRAF mutation inhibitor treatment is considered.

Patient 8 had NRAS mutation Q61K in approximately 94% of tumor cells and BRAF V600E in 4%. Patient 11 had KRAS Q61H mutation in 10% of tumor cells, and a small clone positive for BRAF V600E by IHC that was not detected by WES. These two cases confirm that several mutations in the same signaling pathway can be present in one patient, but not necessarily in the same tumor cells.^11^ Both these patients responded with VGPR.

Several of our observations contrast those of Andrulis *et al.*,^8^ who found a BRAF V600E mutation four times more frequent in EMD compared with bone marrow. They also found significantly shorter OS in patients with BRAF V600E, and reported that the disease seemed to become more aggressive as soon as the BRAF V600E mutation was present. This view is supported by three recently published case reports of very aggressive BRAF V600E mutated myeloma, which quickly became refractory to conventional treatment.^9, 10^ We could not confirm these observations. The differences between the studies may be explained by different selection of patients and random variation. Evidently, patients with BRAF V600E can have aggressive, treatment refractory disease, but also indolent disease as documented in our study.

By this integrated approach to early-stage multiple myeloma, we found that patients with BRAF V600E mutation responded very well to broad acting drugs and there was no relation to prognosis in early stage myeloma. In particular, a large mutated cell fraction did not herald aggressive disease. This study demonstrates that the role of BRAF mutation V600E is more diverse than previously assumed.

## Figures and Tables

**Table 1:** Analysis of 11 patients with BRAF V600E

*Patient no.*	*BRAF V600E fraction IHC (%)*	*BRAF V600E fraction WES (%)*	*BRAF V600E Sanger*	*NRAS/KRAS fraction WES*	*Overall survival (months)*	*Best response to treatment*	*Sensitive, ⩾PR*	*Resistant, <PR*
1	75–100	86	Pos	0	48	VGPR	MP	
1^a^	ND	28	ND	0	48	VGPR	MP	
2	75–100	ND	Neg	ND	77^b^	PR	CP	MP, T, BP, LP
3	75–100	ND	Pos	ND	89	CR	MPT, TD, T	
4	50–75	ND	Pos	ND	11^b^	Missing data	Missing data	Missing data
5	50–75	ND	Pos	ND	51^b^	nCR	MPT, BD, MPB, LD	
6	25–50	ND	Neg	ND	79^b^	VGPR	CD+HDSCT, BD+HDSCT	CBD, L, T
7	25–50	ND	Neg	ND	23^b^	PR	MP, LD	MPT
8	<25	0	Neg	KRAS p.Q61H, 10 %	17	VGPR	CBD+HDSCT	
9	<25	ND	Pos	ND	0^b^	Missing data	Missing data	Missing data
10	<25	ND	Pos	ND	238*	nCR	VAD, VAD+HDSCT, CD, BD, CBD	M2 (M, C, P, V, karmustine)
11	neg	4	Neg	NRAS p.Q61K, 94 %	16	VGPR	MPT, T	None

Abbreviations: A, adriamycine; B, bortezomib; C, cyclophosphamide; D, dexamethasone; HDSCT, high-dose therapy and autologous stem cell transplant; L, lenalidomide; M, melphalan; nCR, near CR (CR not confirmed by bone marrow aspiration); ND, not determined; Neg, negative; Pos, positive; P, prednisone; T, thalidomide; V, vincristine.

All biopsies were from bone marrow with the exception of that from patient 3, which was from an osteolytic lesion in the skull. Biopsies were from relapsed disease for patients 6 and 10, and at time of diagnosis for the remaining patients. Results from IHC are reported as the estimated percentage of CD138 or kappa/lambda-positive cells that are also positive for BRAF V600E.

a Bone marrow aspirate obtained after treatment for first relapse.

b Overall survival is calculated from diagnosis to death (*) or censoring date.

**Table 2:** Clinical characteristics of patients with or without BRAF V600E mutation

	*BRAF V600E positive,* n=*11*	*BRAF V600E negative,* n*=198*	P*-value*
Age at diagnosis^a^, years	69 (27)	68 (18)	0.896
Sex (male)	6/11 (54.5%)	126/198 (64.6%)	0.538
Light chain disease	3/11 (27.3%)	32/187 (17.1%)	0.888
Non-secretory	0/11	3/187 (1.6%)	
IgG	5/11 (45.5%)	96/187 (51.3%)	
IgA	3/11 (27.3%)	46/187 (24.6%)	
IgD	0/11	6/187 (3.2%)	
IgE	0/11	1/187 (0.5%)	
IgM	0/11	3/187 (1.6%)	
Non-secretory	0/11	3/174 (1.7%)	0.375
Kappa	5/11 (45.5%)	112/174 (64.4%)	
Lambda	6/11 (54.5%)	59/174 (33.9%)	
ISS stage 1	2/7 (28.6%)	40/141 (28.4%)	0.891
ISS stage 2	2/7 (28.6%)	54/141 (38.3%)	
ISS stage 3	3/7 (42.9.%)	47/141 (33.3%)	
S-creatinine, μmol/l^a^	125 (463)	86 (48)	0.023
Corrected calcium, mmol/l^a^	2.52 (0.26)	2.48 (0.37)	0.878
Hemoglobin, g/dl^b^	11.9 (2.4)	11.0 (2.1)	0.257
Albumin, g/l^a^	38.0 (10)	35.0 (8)	0.512
Beta-2-microglobulin, mg/l^a^	5.0 (5.4)	4.2 (4.0)	0.907
Bone disease present	9/10 (90%)	105/131 (80.2%)	0.687
EMD	4/11 (36.4%)	72/167 (43.1%)	1
EMD separate from bone	1/11 (9.1%)	25/167 (15.0%)	1
Treated with novel drugs^c^	9/10 (90%)	133/178 (74.7%)	0.455
Treated with HDSCT	3/11 (27%)	72/184 (39%)	0.749

Abbreviations: EMD, extra medullary disease; HDSCT, high-dose therapy and autologous stem cell transplant.

a Parameters who do not follow a normal distribution are presented as median with interquartile range.

b Parameters with a normal distribution are presented with mean and standard deviation.

c Novel drugs include thalidomide, bortezomib, lenalidomide, carfilzomib.

**Table 3:** Summary of WES from three patients positive for BRAF V600E by PCR

	*Patient 1*	*Patient 8*	*Patient 11*
Total single nucleotide variants in exons	145	75	74
Synonymous mutations	33	12	14
Missense mutations	75	36	41
Nonsense mutations	4	4	1
Mutation in untranslated region	24	16	13
Mutations in splice sites	9	7	5
Detection of BRAF V600E mutation	Mutation present in 68/157 reads (43%)	Mutation present in 0/100 reads	Mutation present in 2/99 reads (2%)
Estimated BRAF V600E clone size	86%	0%	4%
Detection of RAS-mutations	None detected	KRAS Q61H mutation present in 7/146 reads (5%)	NRAS Q61K mutation present in 97/205 reads (47%)
Estimated RAS-mutated clone size	0%	10%	94%

The percentages of mutated tumor cells are estimated given that each cell contains one copy of the mutated gene and that purity of isolated tumor cells is near 100%.

## Notes

Dr Hovig is a shareholder in PubGene Inc., and a board member of GeneSeque AS. Mr Vodák has received a doctoral student grant from PubGene AS. The remaining authors declare no conflict of interest.
